# Quality scores for 32,000 genomes

**DOI:** 10.1186/1944-3277-9-20

**Published:** 2014-12-08

**Authors:** Miriam L Land, Doug Hyatt, Se-Ran Jun, Guruprasad H Kora, Loren J Hauser, Oksana Lukjancenko, David W Ussery

**Affiliations:** 1 Comparative Genomics Group, Biosciences Division, Oak Ridge National Laboratory, P.O. Box 2008, MS 6420, Oak Ridge, TN 37831-6420, USA; 2 Joint Institute for Biological Sciences, University of Tennessee, Knoxville, TN 37996, USA; 3 Computer Science and Mathematics Division, Computer Science Research Group, Oak Ridge National Laboratory, Oak Ridge, TN 37831, USA; 4 Department of Microbiology, University of Tennessee, Knoxville, TN 37996, USA; 5 Center for Biological Sequence Analysis, Department of Systems Biology, The Technical University of Denmark, Kgs. Lyngby 2800, Denmark; 6 Center for Genomic Epidemiology, Technical University of Denmark, Kongens Lyngby 2800, Denmark

**Keywords:** DNA, Sequencing, Database, Quality, Evaluation, Status

## Abstract

**Background:**

More than 80% of the microbial genomes in GenBank are of ‘draft’ quality (12,553 draft vs. 2,679 finished, as of October, 2013). We have examined all the microbial DNA sequences available for complete, draft, and Sequence Read Archive genomes in GenBank as well as three other major public databases, and assigned quality scores for more than 30,000 prokaryotic genome sequences.

**Results:**

Scores were assigned using four categories: the completeness of the assembly, the presence of full-length rRNA genes, tRNA composition and the presence of a set of 102 conserved genes in prokaryotes. Most (~88%) of the genomes had quality scores of 0.8 or better and can be safely used for standard comparative genomics analysis. We compared genomes across factors that may influence the score. We found that although sequencing depth coverage of over 100x did not ensure a better score, sequencing read length was a better indicator of sequencing quality. With few exceptions, most of the 30,000 genomes have nearly all the 102 essential genes.

**Conclusions:**

The score can be used to set thresholds for screening data when analyzing “all published genomes” and reference data is either not available or not applicable. The scores highlighted organisms for which commonly used tools do not perform well. This information can be used to improve tools and to serve a broad group of users as more diverse organisms are sequenced. Unexpectedly, the comparison of predicted tRNAs across 15,000 high quality genomes showed that anticodons beginning with an ‘A’ (codons ending with a ‘U’) are almost non-existent, with the exception of one arginine codon (CGU); this has been noted previously in the literature for a few genomes, but not with the depth found here.

## Background

The introduction of second-generation sequencing began an exponential growth in sequencing data [1-4] and in the number of genomes submitted to public repositories. The drop in sequencing cost that came with this technology, however, had little effect the mostly manual cost of finishing genomes. Finishing second-generation sequenced genomes continues to be expensive and many researchers have no plans to finish their draft genomes [5]. There is still an open question of whether whole genome sequencing projects with less than 5% of the genes missing is adequate quality for most purposes [6] or if there continues to be value in finishing most microbial genomes [7]. Even though single molecule, or ‘third-generation’ sequencing will facilitate the generation of closed genomes, currently most of the genomes in the database are of varying levels of draft quality.

The establishment of a quality nomenclature by Chain *et al.* in 2009 [8] provides a mechanism for comparing draft sequences and understanding the qualifiers associated with a single genome sequence. It does not, however, shine any light on the impact that predominately draft genomes have on the quality of the repository databases. With more than 30,000 unique publicly available genome sequences of varying qualities, there is enough data to score genomes on the basis of completeness and compare quality among data sources.

DNA sequences were obtained from two sources at GenBank and the National Center for Biotechnology Information [9]: draft genomes (WGS or ‘draft’) and complete finished genomes (‘complete’). An assembled version of the GenBank Sequence Read Archive was obtained for analysis [10]. Despite major overlaps, three additional data sources, the Broad Institute [11], Pathosystems Resource Integration Center [12], and the United States Department of Energy (DOE) Systems Biology Knowledgebase (http://kbase.us), were acquired because they contained additional unique genome sequences. The DNA sequences were then scored for completeness.

Estimates of genome quality were based on 1.) the sequence quality (number of contigs and number of non-standard bases); 2.) the presence of a full-length 5S, 16S, and 23S rRNA; 3.) the presence of at least one tRNA coding for all of the 20 standard amino acids; and 4.) the presence of a set of essential genes containing 102 conserved Pfam-A [13] domains found in nearly all bacteria and archaea (Additional file 1: Table S1). Software tools were either selected or developed to provide an estimate for each of these measures of completeness. The four individual scores ranged from zero to one and they were averaged for a combined score. The data sources and calculation of the scores are described in more detail in the Methods section.

To keep all scores comparable, we ran standard predictions using the same settings across all genomic DNA sequences; tRNAscan-SE [14] was used to predict tRNAs, RNAmmer [15] was used to predict rRNAs, and Prodigal [16] was used to predict protein coding genes in all the acquired sequences. HMMER3 [17] was used to find Pfam-A [13] domains. We chose not to use the predictions from the source databases because consistent annotation was not available for all sequences and the resultant scores would have been a reflection of the source annotation and not just the completeness of the sequence. A score for annotation quality may be added to future versions of this scoring system.

## Results and discussion

**Figure 1:**
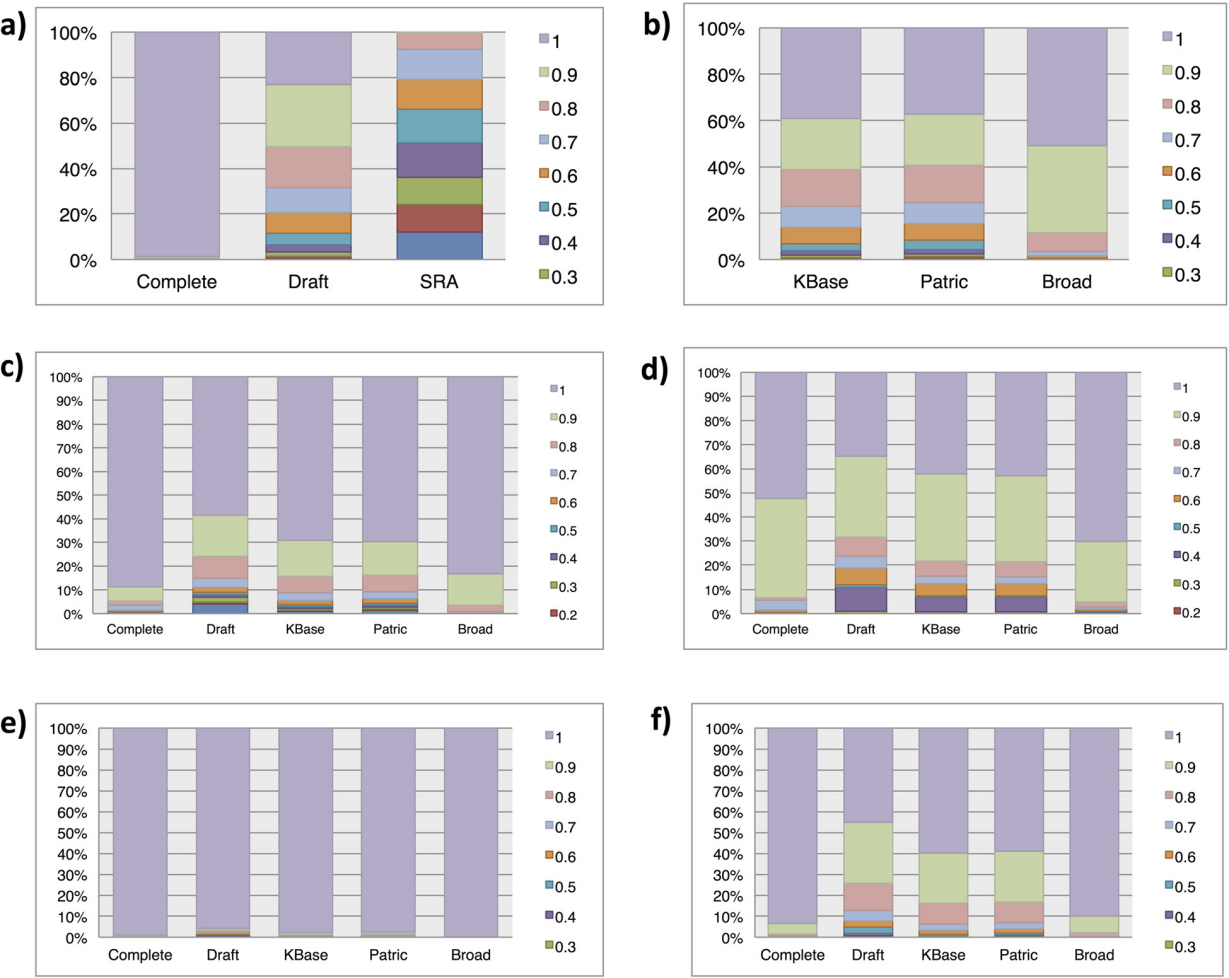
**Comparison of quality scores between the data sources.** For each data source, the percent of genomes within each range of scores. The number in the legend is the largest value in the range. Ranges with no genomes are not presented in the legends. The six tables are scores for **(a)** Sequence quality from GenBank Sources, **(b)** Sequence quality from Non-GenBank Sources, **(c)** tRNAs (one each of 20 standard amino acids), **(d)** rRNAs (one full size 5S, 16S, and 23S rRNA), **(e)** Essential Genes (102 conserved Pfam-A domains) and **(f)** Total combined Scores.

The number of genomes found in each source varied from 12,553 in the WGS genomes to 2,477 in the Broad. There were 20,367 genomes only found in a single source and 11,696 from more than one source for a total of 32,063 unique genome assemblies using the MD5 checksum method of determining uniqueness (Table 1). Most data sources had at least one internal duplicate using the MD5 checksum.As expected, among GenBank genomes, the ‘complete’ genomes tended to score the best, followed by the WGS genomes and lowest quality scores were for the assemblies of SRA reads (Figure 1). In the genomes from PATRIC, Broad, and KBase databases, it was more difficult to separate draft and finished genomes. Their scores therefore are similar to a mixture of the ‘draft’ and ‘complete’ genomes at NCBI. One counterintuitive result was the existence of a handful of ‘complete’ genomes with relatively low scores, and a small set of ‘draft’ genomes that received perfect scores.

**Table 1:** Counts of total and unique genomes acquired from each data source

**Data source**	**Count**	**Unique to this source**
GenBank complete finished	2,679	500
GenBank draft	12,553	4,759
GenBank SRA - assembled	11,767	11,750
PATRIC	12,245	771
DOE KBase	11,944	396
Broad	2,477	2,191
Total	53,665	20,367

As noted in the discussion, factors other than data source can influence the score. With over 20,000 genomes, it is possible to analyze some of these factors and highlight a few intriguing observations.

### Sequence quality score

The sequence quality is a function of the number of contigs per megabase (counting N's as gaps) and the number of non-standard bases per genome. The sequence quality scores varied between the different sources (Figures 1a and b and Additional file 1: Table S2). Surprisingly, about 2% of the ‘complete’ genomes did not have perfect scores, while 3% of the WGS genomes received perfect scores (0.99 or better), despite being ‘draft’. As might be expected, the collection of SRA genomes had lower quality scores, with a maximum sequence quality of 0.88 and an average of 0.38. All other databases had an average score of 0.75 or better. The SRA genomes scored low enough on sequence quality that additional analysis was not done on these genomes.

The number of contigs per genome ranged from 1 to 13,915 (Additional file 2: Figure S1 and Additional file 1: Table S3). The average for the ‘complete’ genomes was about 5 replicons per genome and the average contig counts for ‘draft’, KBase, PATRIC, and Broad were 190, 130, 151, and 48, respectively. The results included a ‘complete’ genome with 930 contigs and 70 ‘draft’ genomes that contained a single contig/scaffold.

### tRNA score and anticodons

Most genomes scored well with respect to having at least one tRNA codon for each amino acid (Figure 1c and Additional file 1: Table S4). Even among the WGS genomes, nearly 60% of the genomes had perfect scores for tRNA genes (all 20 types). As expected, the ‘complete’ genomes scored better than other data sources, although 7 of these genomes had a score of 0.1 (9 or more missing tRNAs). Among the low-scoring ‘complete’ genomes was *Pyrobaculum calidifontis* JCM 11548, *Thermoproteus uzoniensis* 768-20, and two “*Candidatus* Tremblaya princeps”. Factors that may contribute to a low score are: 1) *P. calidifontis* and *T. usoniensis* are part of the 0.1% of genomes that have 9 or more predicted pseudo tRNA genes not included in the calculations and 2) the *Candidatus* genomes are endosymbionts with less than 140 kbp DNA in their chromosome.

The number of tRNAs per genome were compared across the data sources (Additional file 2: Figure S2 and Additional file 1: Table S5). These did not affect the score but provided some interesting observations. The maximum number found was 280 for *Escherichia coli* HVH 33 (4-2174936). This genome was found at both Broad and WGS and the number of tRNAs was high compared to the average of 79 +/- 13 for the other 1500 *E. coli* genomes. The number of rRNA molecules for this genome is in line with other *Escherichias* (5 23S and 7 16S), and it is likely that this is a single genome.

**Figure 2:**
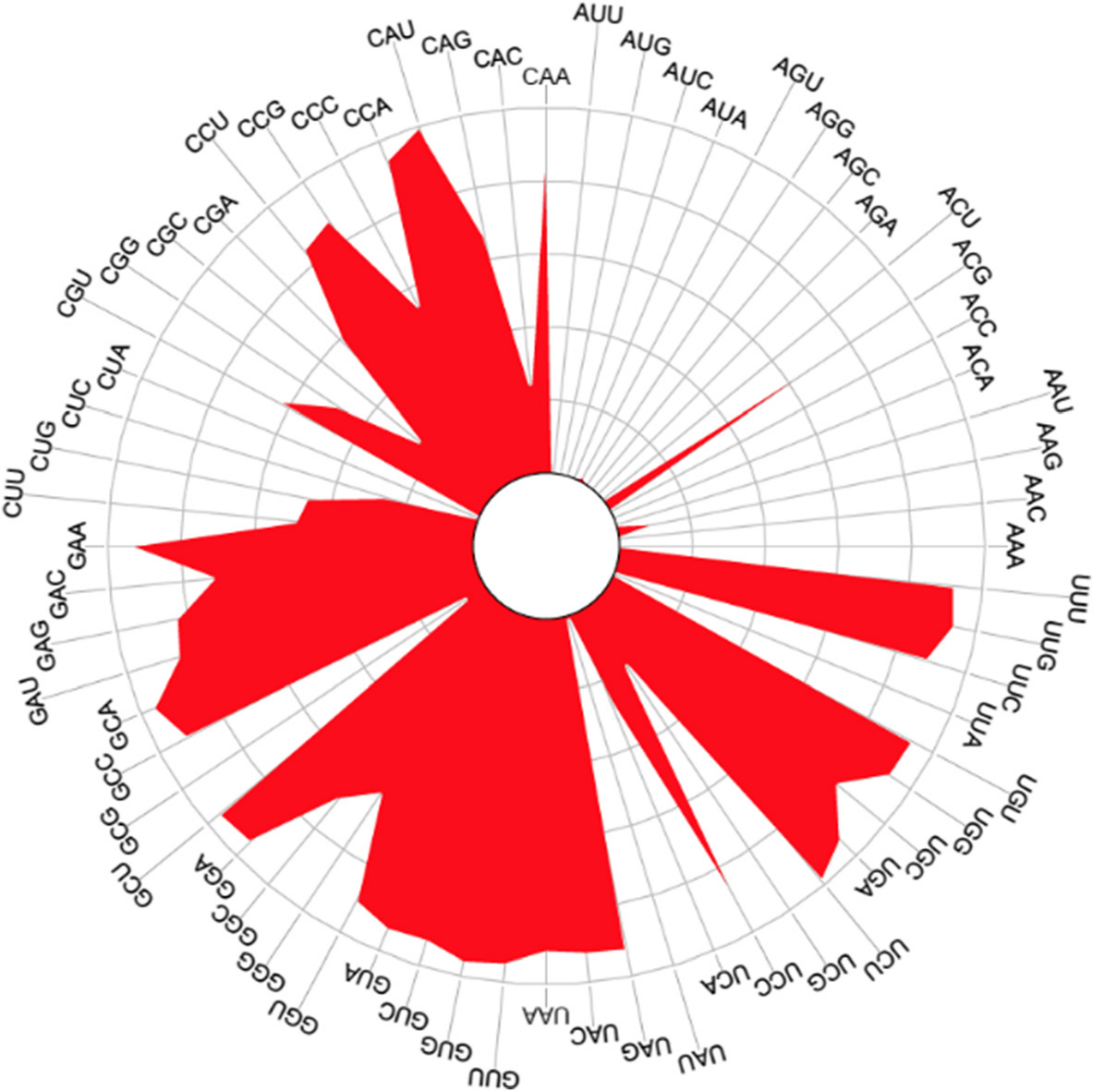
**Rose plot of predicted tRNA anticodon frequency.** Length of line from center outwards indicates relative frequency. Each quadrant corresponds to a different starting base for the anticodon. The upper right quadrant contains the anticodons that start with ‘A’ and are relatively rare.

While it is possible for a genome to have tRNAs with up to 62 different anticodons, surprisingly, the maximum found, out of more than 20,000 genomes, was only 47 (Additional file 2: Figure S3 and Additional file 1: Tables S6). The average number for all data sources was between 33 and 37. Of the 62 possible anticodons, 15 of the 16 anticodons starting with an A (codon ending with U) were relatively rare (Figure 2). For example, the anticodon ACA was only detected 8 times (Additional file 1: Table S7). The anticodon ‘ACG’ (arginine) was the only one predicted in a large number of genomes. This is consistent with findings from other researchers [18], although this is the first time this observation has been made with such a large number of bacterial genomes.

A list of rarely predicted tRNAs by genus (Additional file 1: Table S8) shows that rare anticodons are over-represented in some genera. The percent of genomes with rare anticodons in the genera *Escherichia, Butyrivibrio* and *Lactobacillus* were compared (Table 2). Only 0.1% *Escherichia* genomes in our analysis have a rare tRNA. In contrast, 87% and 44% of *Butyrivibrio* and *Lactobacillus,* respectively, have genomes with a rare tRNA. This implies that part of the bias observed might be reflective of the large presence of *E. coli* and other common organisms that are easy to grow and cultivate in the lab.

**Table 2:** Comparison of 3 selected genera and the number of genomes with rare anticodons found in the analysis

**Genus**	**Number in study**	**Number with rare anticodons**	**Percent**
*Butyrivibrio*	32	28	87
*Escherichia*	2177	3	0.1
*Lactobacillus*	338	150	44

### rRNA score and length distribution

The lengths of the rRNA molecules were compared across data sources (Additional file 2: Figures S4-S7 and Additional file 1: Tables S10-S13). In our scoring scheme, the lowest possible score for rRNAs was 0.1. All genomes scored 0.3 or better (Figure 1d and Additional file 1: Table S9) and 5.7% of the ‘complete’ genomes scored below 0.9.

Some of the extremely long 16S predictions were investigated. The predictions of 7 genomes with extremely long rRNA genes were compared to the annotations at GenBank. Two had the same predictions in GenBank, four had more reasonable predictions at GenBank, and one genome was missing all 16S predictions, including an abnormally long one predicted by RNAmmer.

Most genomes with abnormally long 16S rRNA (greater than 2300 bases) fell into one of the following categories, 1) the DNA encoding the rRNA gene contained Ns, 2) the genome contained one or more “normal” predictions, or 3) the genome was less than 40% GC. The first is an indicator of a problem in the sequencing, the second may indicate an atypical region of the genome, and the third may be a weakness of the RNAmmer tool.

### Essential gene score

Protein-coding genes were predicted for all genomes using Prodigal [16] and average gene length and density were calculated (Additional file 2: Figures S8-S9 and Additional file 1: Tables S14-S15). The average gene length was expected to be slightly less than 1000. The Broad genes had the longest average length at 940 bases and the PATRIC had the shortest average length at 902 bases. WGS contained the individual genome with the shortest average length (200 bases) while WGS, KBase, and PATRIC all had the genome with the longest average length (1291 bases).

The distribution of essential gene scores shows that all of the data sources are very similar (Figure 1e and Additional file 1: Table S16). The set of ‘universally conserved domains’ was surprisingly well conserved, being found in nearly all of the more than 20,000 genomes. The percent of genomes with perfect scores of 1 ranged from 96% for WGS to 99.9% for Broad. The Pfam-A model that was missed the most often is the 60-65 residue ribosomal protein S14p. This may be a reflection of the inability of the gene finder to find the genes rather than missing domains. The protein predictions for *Clostridium clariflavum* DSM 19732 *and Clostridium thermocellum* DSM 1313 were each missing a S14p domain-containing protein. A 6-frame translation search of the genomes revealed that a sequence matching the model was in the DNA.

### Total combined score

The total score was calculated by averaging the other normalized four scores (Figure 1f and Additional file 1: Table S17). The ‘complete’ genomes had an average score of 0.97 and the average score was 0.85 for the WGS draft genomes. Although only 6% of the genomes had a perfect quality score, most (~88%) of the genomes had quality scores of 0.8 or better. At the other extreme, about 3% of the genomes had a score below 0.6 and probably have too low a quality to yield reliable analysis. This score corresponds to more than 1000 contigs.

While the ‘complete’ genomes were the best scoring on average, there are a few low quality genomes in this database. Among them are *Borrelia valaisiana* VS116 (0.27) and *Bacillus anthracis* str. A2012 (0.27). The draft WGS genomes on average have lower scores, although a 28 genomes in the WGS dataset scored a perfect 1.0. To achieve a perfect score, the sequence must be in one contiguous piece and contain no runs of ‘N’ greater than 10 bases.

The data, algorithm and score cards for all the genomes are accessible from our website [19]. The results of the study can be downloaded from the results page of our website.

The data were examined to identify underlying factors that may have contributed to the score. From GenBank files and the PATRIC web site it was possible to gather the sequencing technology, the assembly method, coverage, and update date for many of the genomes. From Broad it was possible to gather a sequencing technology and coverage for many genomes. It was not possible to entirely account for the effect of read length, experience of the researchers, all version changes, the wide disparity in the number of available genomes, or the fact that the information was not available for most early genomes. Care should be used when drawing conclusions from the data.

The analysis by date showed that older genomes were predominately complete and tended to score better than newer draft genomes (Additional file 2: Figure S10). The data is consistent with graphs showing the differences between complete and draft genomes (Figure 1).

**Figure 3:**
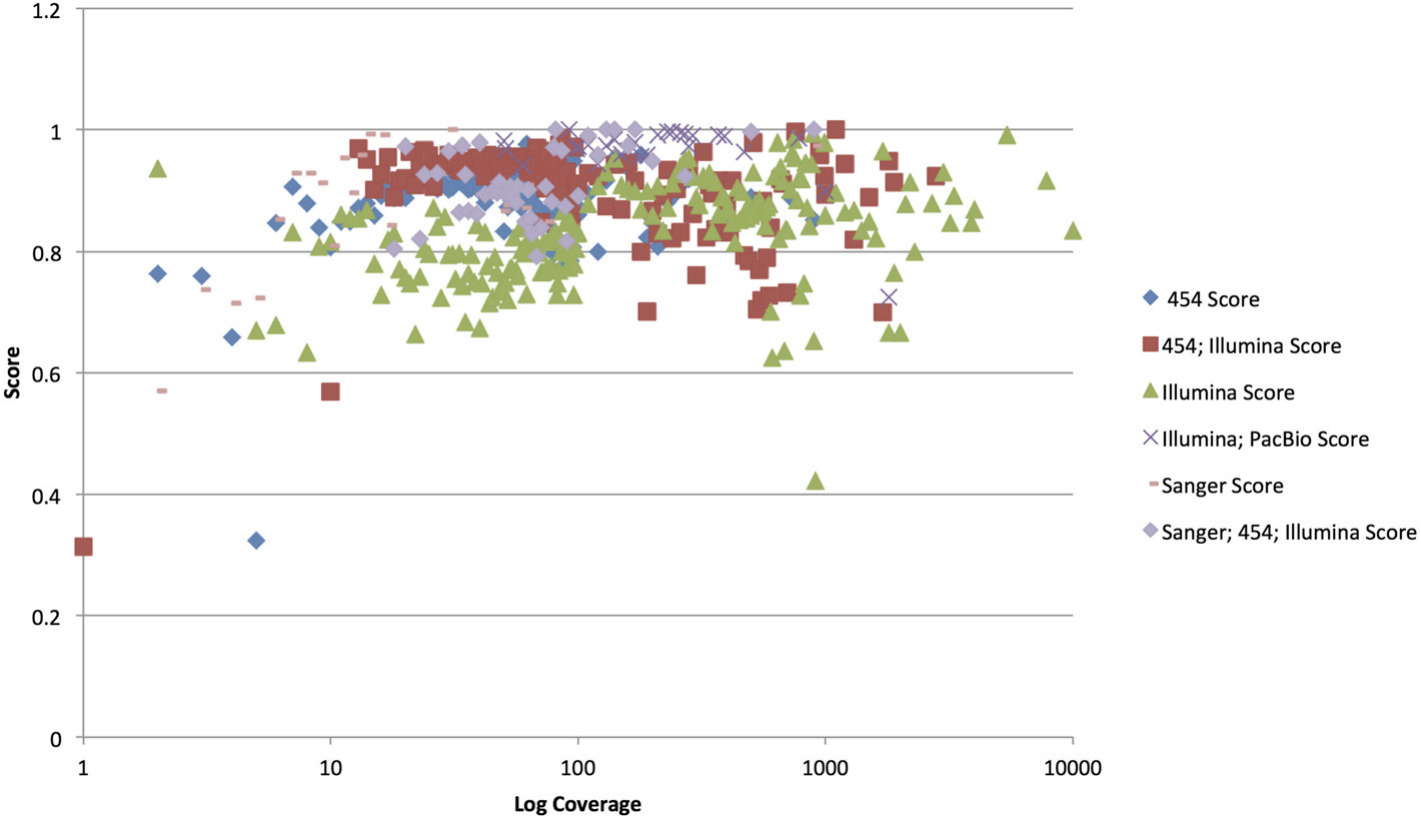
**Plot of average score by log coverage level for each sequencing technology.** The coverage level and sequencing technology extracted from GenBank and PATRIC sources. The log of the coverage is plotted by score and symbols assigned by sequencing technology. Due to the density of the data in the plot, less frequent sequencing technologies are not shown.

The analysis by percent GC and genome size only suggested that larger assemblies are more likely to have all the necessary components (Additional file 2: Figure S11) and percent GC is not a determining factor (Additional file 2: Figure S12). The analysis by coverage did not show any differences until sequencing technology was taken into consideration (Figure 3). It shows that when using 454, Illumina, or a mixture of the two, coverage of over 100× did not necessarily lead to a better scores and sometimes it was worse. It also shows that a combination of Illumina and PacBio often scored well, up to a coverage level of about 1000 and then dropped off.

**Figure 4:**
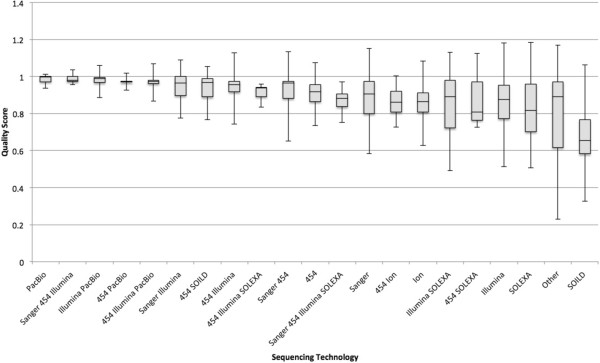
**Box and whiskers plot of average score by sequencing technology.** Where available, a sequencing technology was parsed from GenBank and PATRIC sources. Data are sorted left to right from largest to smallest mean value. The box represents the first quartile, the mean, and the third quartile. The whiskers represent 2 standard deviations on either side of the mean. Because the data have an upper limit of 1, the upper range can exceed the possible values.

**Figure 5:**
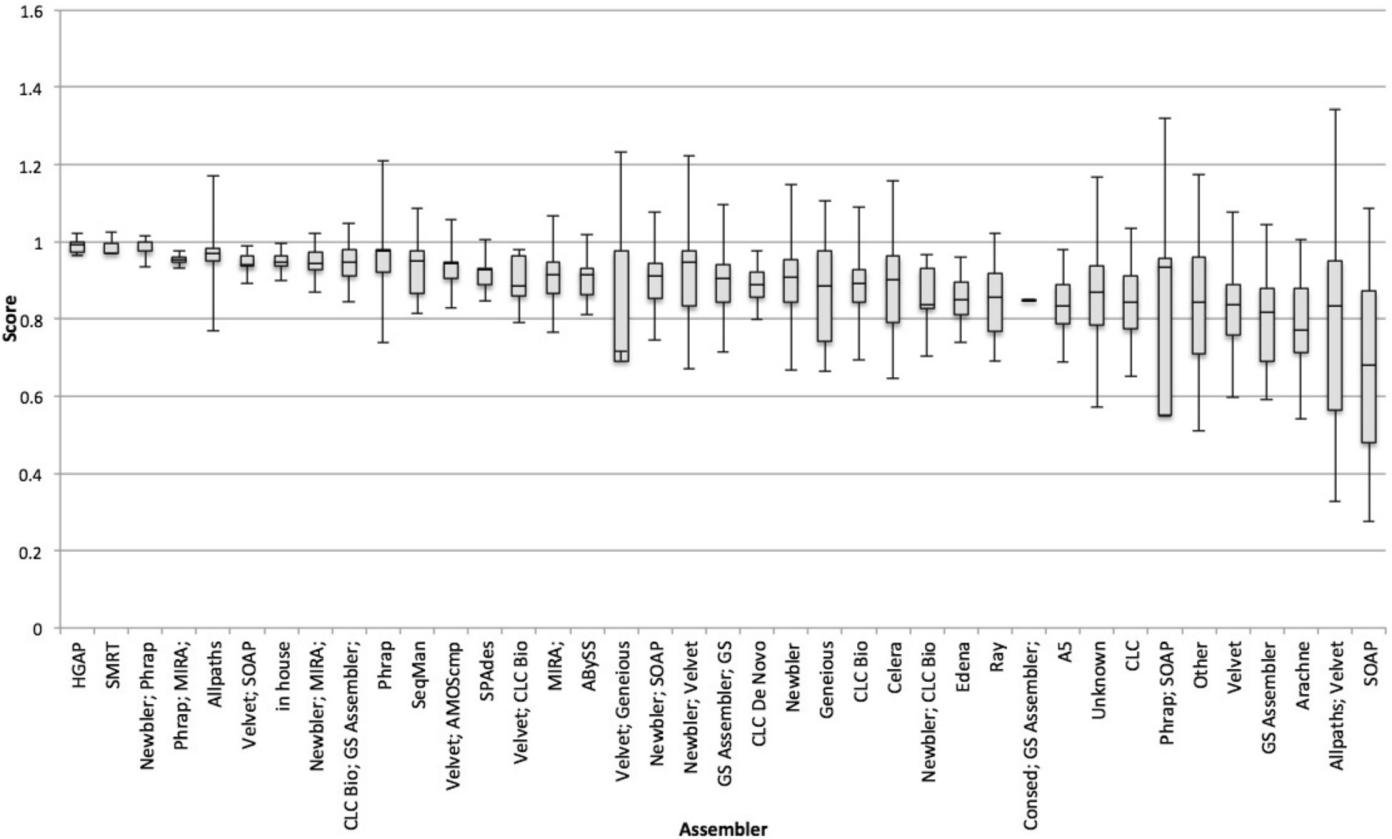
**Box and whiskers plot of average score by assembler.** Where available, a assembly method was parsed from GenBank and PATRIC sources. Data are sorted left to right from largest to smallest mean value. The box represents the first quartile, the mean, and the third quartile. The whiskers represent 2 standard deviations on either side of the mean. Because the data have an upper limit of 1, the upper range can exceed the possible values.

An analysis was done by sequencing technology and assembly method (Figures 4 and 5 and Additional file 1: Tables S18 and S19). The primary observation is that the small number of genomes using PacBio as one of its sequencing technologies have done well so far and SOLID has scored lower. Because PacBio has a reputation for longer reads of inaccurate quality [20] one possible interpretation is that read length affects quality score more than sequencing quality. Time will tell whether or not this continues to hold true over thousands of genomes.

**Figure 6:**
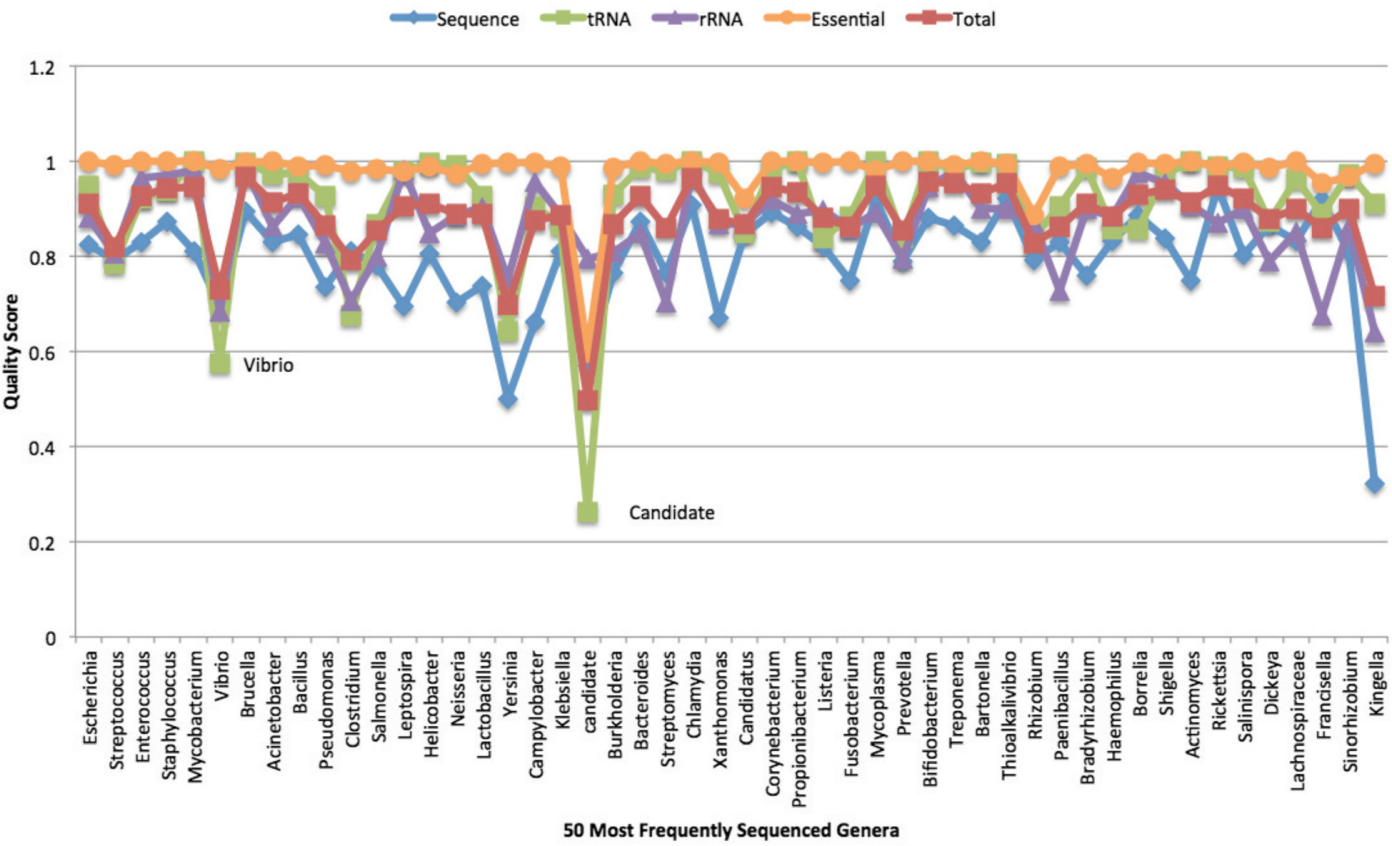
**Quality scores for 50 most abundant genera.** Average quality scores for sequence, tRNAs, rRNAs, essential genes, and total plotted for each of the 50 most represented genera. The genera are presented in order of abundance from Escherichia on the left to Kingella on the right.

Scores were compared by genus (Figure 6). Only the 50 most abundant genera are presented and they are listed left to right by most abundant (2170 genomes) to least abundant (49 genomes). Except for Candidatus and “candidate division”, most genera have pretty good average scores for the essential genes. This is despite the fact that there is a large percent of draft genomes in the analysis. The interesting exception is *Rhizobium*.

In several histograms, Broad appears to be an outlier. For example, in Additional file 2: Figure S9, Broad has a higher percentage of genomes between .9 and 1.0 genes per kilobase than any other source. This was investigated and the taxonomic makeup of the Broad is also an outlier. Eighty percent of the Broad genomes belong to 8 genera (*Escherichia, Enterococcus, Staphylococcus, Brucella, Acinetobacter, Mycobacterium, Ba.cil.lus* and *Pseudomonas*), compared to 34% in other sources (Additional file 1: Table S20).

## Conclusions

A final conclusion from this scoring review is that widely used analysis tools performed well most of the time, but each had a point where they seemed to miss the mark. tRNAscan predicted pseudogenes rather than real genes in some genomes, RNAmmer predicted unrealistically long genes under some circumstances, and Prodigal occasionally missed a few valid small genes instead of lots of small false-positive genes.

The normalization process did not change the scores by very much. Each investigator’s preferences for a score cutoff will have greater impact on the assignment of a “good” vs a “bad” score. For future use, it is recommended that the standardization step be skipped for analysis of a single genome.

Automated annotation should be checked for the minimal quality components presented here. The annotation may need to be manually edited to compensate for the rare occasions when the tools give misleading or missing results.

Genome sequences are used by researchers in several fields to answer many different types of scientific questions. The score presented in this work is one metric among many that can be used even though none are suitable in all circumstances. For example, when comparing assembly methods and/or strains within the same species, well established measures such as N50 or use of a reference sequence will be more targeted and specific than a single score. Also, the score cannot address errors associated with sample preparation, contamination, or misidentification of the genus, species and/or strain. A very high quality sequence can lead to a flawed analysis if it is incorrectly identified.

## Methods

### Obtaining the data

Complete finished GenBank genomes were obtained from the NCBI ftp site [21]. GenBank and Fasta files were extracted from each subdirectory.

Draft GenBank genomes were obtained from the NCBI WGS ftp directory [22]. All projects labeled as belonging to the BCT division were downloaded and GenBank and Fasta files were stored for each of them.

Broad genomes were obtained from the Broad Olive web site [11]. Fasta files were downloaded for each Broad project. Genomes were divided by sequencing status.

PATRIC genomes were obtained from the PATRIC ftp site [23]. Fasta files were downloaded for each PATRIC genome. Genomes were divided by sequencing status.

SRA: genomes were downloaded from the NCBI genomes SRA [24] in January 2012, and assembled as described previously by Larsen *et al.* 2012 [10]. All genomes were assumed to be draft.

KBase genomes were downloaded using the API commands all_entities_Genome to get the master list of Bacterial and Archaeal genomes. The API commands genomes_to_contigs and contigs_to_sequences were used to download the sequences for the genomes. KBase does not provide a sequencing status for genomes.

Additional data repositories could have been considered for this paper. It was assumed that repositories that attempt to include “all possible genomes” would have largely overlapping sets of genomes. This was supported by the data in Table 1. Only the data sets with a unique focus, such as the Sequence Read Archive and the Broad (selected genera), had a large percentage of unique genomes. The scoring system could be applied to any repository that routinely finds rRNAs, tRNAs, and functional domains for its genomes.

### Definition of unique genomes

Identification of different assemblies for the same genome sequence is difficult, as strain meta-data can be missing or slightly different. For this analysis it was necessary to automatically determine unique genome sequences, due to the number of genomes. Name matching is problematic because names change with time and data sources can represent strain names with different syntax. Even if the data sources had names of other identifiers that linked their data to one or more of the other sources, there is the issue of ensuring they are the same version of the genome.

While not perfect, an MD5 checksum algorithm was used to identify duplicate assemblies. Minor differences in assemblies or treatment of gaps produce different checksums and therefore two genomes that are for all practical purposes the same, appear to be different. Most data sources had at least one internal duplicate using the MD5 checksum.

The MD5 hex checksum for a genome was calculated by first creating an MD5 checksum of all component contigs. These checksums were sorted and concatenated into a new string with comma separators and no spaces. The checksum of the genome was the checksum of this new string.

Genomes were sorted by size and the names of the largest and smallest genomes were examined. Genomes with a total size of less than 138,500 bp were found to be plasmids and genomes greater than 18,000,000 bp were found to be eukaryotic. The plasmids and eukaryotic genomes were deleted from the databases. One GenBank genome was eliminated because it was a project containing a set of 10 different genomes, rather than representing a single genome.

### How the scores are assigned, by component

Calculation of quality scores was performed in two stages. In the first stage, algorithms were run to determine the range of values associated with a metric (*e.g.*, the number of ‘essential genes’ found for a given genome). In the second stage, a score was scaled to represent our assessment of the quality of that level of completeness. For example, a score of 0.9 was assigned if 90% of essential genes were found.

*The sequence quality score* included a combination of contigs and non-standard bases. Large numbers of contigs are an indicator of an incomplete genome. Strings of N’s over 10 bp were assumed to be gaps and added the same penalty as an additional contig. The score was calculated as a fraction using:

1. The numerator was the number of “good” bases across all contigs (i.e., A, C, T, or G).

2. The denominator was the number of “good” bases, plus

o the count of “bad” (bad = anything except A, C, T, G, or N) bases, plus

o a 10,000 bp “penalty” bases for each contig after the first, plus

o a 10,000 bp “penalty” for each gap of 10 or more N's

3. ‘Complete’ genomes were penalized for gaps of 10 or more N's but were not penalized for additional contigs, which were assumed to be additional plasmids or chromosomes.

The sequence quality score was designed to estimate how close the genome was to being completely sequenced – that is, once contiguous piece per replicon. In principle, this would likely be reflective of the reliability of coverage – that is, that enough of the genome is present in good enough quality so as to minimize errors in the prediction of all the genes and features by protein-coding and RNA gene prediction algorithms. Gene prediction algorithms may fail to predict genes in extremely short contigs, or at the edges of longer contigs. It was generally assumed that each contig would lose, on average, half a gene at each edge, or one gene total, with an average size of 900-1000 bp. However, we did not consider an assembly that could only capture 90% of genes to be of high quality, so we scaled the score downward by increasing this “missing bases” penalty by a factor of 10.

*The rRNA score* was calculated using the 5S, 16S, and 23S rRNA predictions from RNAmmer version 1.2 [15]. A minimum and maximum length was established for each molecule type by plotting the distribution of all lengths and picking a value where the distribution dropped off dramatically (data not shown). The 23S has a broader length range to accommodate the molecules with introns. The score was calculated as follows:

1. Start with a minimum of 0.1.

2. Defined an ideal length range for each molecule type:

a. 23S to be between 2900 and 3500

b. 16S to be between 1450 and 1700

c. 5S to be between 100 and 120

3. For each molecule type,

a. add .3 if length was within the ideal range

b. else, add .2 if length was greater than 0.5 times the minimum

c. else, add .1 if a prediction of any length exists

*The tRNA score* was based on tRNAscan-SE 1.3.1 [14] and predictions for at least one tRNA that coded for each of the 20 standard amino acids. Because the tRNAs are highly conserved and relatively easy to locate, genomes with 10 or more missing amino acids was determined to be very low quality and got the lowest possible score. The optional tRNAs (e.g., selenocysteine) were included in the count of total tRNAs and total anticodons. The score was calculated as follows:

1. Start with a maximum of 1.0

2. Subtract 0.1 for each amino acid with no tRNA, until a minimum of 0.1 is reached.

*Essential genes* were defined as 102 Pfam-A domains found to be present in nearly all bacterial and archaeal genomes (Additional file 1: Table S1). The domains were determined by scanning 2010 complete genomes with Pfam-A and selecting those domains that were present in 99% of the genomes. The data set included 1982 bacterial and 128 archaeal genomes available from GenBank in September 2012. Prodigal [16] was used to predict the genes and obtain translations for each genome. Prodigal has a known weakness for occasionally missing one or two of the small genes in the list of essential genes. It was used because it provided a consistent basis for comparing all of the genomes and the missing genes would be in the noise at a whole database scale. HMMER3 [17] was then used to find the 102 essential Pfam-A models in the gene predictions. The score was assigned as follows:

1. Start with a maximum of 1.0

2. Subtract 0.01 for each missing Pfam-A until a minimum of 0.1 is reached.

The four individual scores were each standardized to a zero mean and unit variance (x_new_ = (x-μ)/σ) and then averaged. The average was transformed to a scale with a minimum of zero and a maximum of one. The total combined score is this transformed value.

Numbers in all the Supplementary tables were rounded to two significant digits. Some integers presented in the text (e.g., maximum length of 23S), are given with more digits of precision.

## Competing interests

The authors have no competing interests.

## Author contributions

DWU conceived the idea. DWU, MLL, PDH, SRJ, and LJH designed the scoring methodology. MLL, PDH, OL, and SRJ analyzed the data. GHK implemented the website. MLL wrote the manuscript with the assistance of the other authors. All authors read and approved the final manuscript.

## Supplementary Material

Additional file 1 Additional tables mentioned in the text with frequency distributions and statistics supporting the analysis. Click here for file

Additional file 2 Additional figures mentioned in the text with bar charts used to visually support the analysis. Click here for file
