# Supplementary material for: Quality scores for 32,000 genomes
Source: Stand Genomic Sci. 2014 Dec 8;9:20. doi: 10.1186/1944-3277-9-20 (PMC4334873; doi:10.1186/1944-3277-9-20)
Supplement: Additional file 1 — Additional tables mentioned in the text with frequency distributions and statistics supporting the analysis. [file 1944-3277-9-20-S1.doc]

**Additional file 1: Tables**

Table S1. One hundred two Essential Genes identified by Pfam-A domain

| **Pfam-A Domain** | **Description** |
| --- | --- |
| PF00344 | SecY translocase |
| PF00004 | ATPase family associated with various cellular activities (AAA) |
| PF00814 | Glycoprotease family |
| PF04563 | RNA polymerase beta subunit |
| PF04997 | RNA polymerase Rpb1 domain 1 |
| PF00623 | RNA polymerase Rpb1 domain 2 |
| PF04983 | RNA polymerase Rpb1 domain 3 |
| PF04998 | RNA polymerase Rpb1 domain 5 |
| PF04565 | RNA polymerase Rpb2 domain 3 |
| PF00562 | RNA polymerase Rpb2 domain 6 |
| PF04560 | RNA polymerase Rpb2 domain 7 |
| PF01193 | RNA polymerase Rpb3/Rpb11 dimerisation domain |
| PF01000 | RNA polymerase Rpb3/RpoA insert domain |
| PF01926 | 50S ribosome-binding GTPase |
| PF03129 | Anticodon binding domain |
| PF08264 | Anticodon-binding domain of tRNA |
| PF00679 | Elongation factor G C-terminus |
| PF03764 | Elongation factor G domain IV |
| PF03144 | Elongation factor Tu domain 2 |
| PF00009 | Elongation factor Tu GTP binding domain |
| PF01336 | OB-fold nucleic acid binding domain |
| PF00673 | Ribosomal L5P family C-terminus |
| PF00466 | Ribosomal protein L10 |
| PF00572 | Ribosomal protein L13 |
| PF00252 | Ribosomal protein L16p/L10e |
| PF00828 | Ribosomal protein L18e/L15 |
| PF00687 | Ribosomal protein L1p/L10e family |
| PF00237 | Ribosomal protein L22p/L17e |
| PF00276 | Ribosomal protein L23 |
| PF00297 | Ribosomal protein L3 |
| PF00573 | Ribosomal protein L4/L1 family |
| PF00281 | Ribosomal protein L5 |
| PF00338 | Ribosomal protein S10p/S20e |
| PF00411 | Ribosomal protein S11 |
| PF00164 | Ribosomal protein S12 |
| PF00416 | Ribosomal protein S13/S18 |
| PF00312 | Ribosomal protein S15 |
| PF00366 | Ribosomal protein S17 |
| PF00203 | Ribosomal protein S19 |
| PF00318 | Ribosomal protein S2 |
| PF00189 | Ribosomal protein S3 C-terminal domain |
| PF03719 | Ribosomal protein S5 C-terminal domain |
| PF00177 | Ribosomal protein S7p/S5e |
| PF00410 | Ribosomal protein S8 |
| PF00380 | Ribosomal protein S9/S16 |
| PF03947 | Ribosomal Proteins L2 C-terminal domain |
| PF00181 | Ribosomal Proteins L2 RNA binding domain |
| PF01479 | S4 domain |
| PF01176 | Translation initiation factor 1A / IF-1 |
| PF11987 | Translation-initiation factor 2 |
| PF00587 | tRNA synthetase class II core domain (G H P S and T) |
| PF00133 | tRNA synthetases class I (I L M and V) |
| PF00579 | tRNA synthetases class I (W and Y) |
| PF00152 | tRNA synthetases class II (D K and N) |
| PF01409 | tRNA synthetases class II core domain (F) |
| PF00005 | ABC transporter |
| PF00266 | Aminotransferase class-V |
| PF00557 | Metallopeptidase family M24 |
| PF07992 | Pyridine nucleotide-disulphide oxidoreductase |
| PF00070 | Pyridine nucleotide-disulphide oxidoreductase |
| PF00696 | Amino acid kinase family |
| PF05746 | DALR anticodon binding domain |
| PF00271 | Helicase conserved C-terminal domain |
| PF13393 | Histidyl-tRNA synthetase |
| PF00861 | Ribosomal L18p/L5e family |
| PF00831 | Ribosomal L29 protein |
| PF00298 | Ribosomal protein L11 RNA binding domain |
| PF00238 | Ribosomal protein L14p/L23e |
| PF00347 | Ribosomal protein L6 |
| PF00333 | Ribosomal protein S5 N-terminal domain |
| PF01751 | Toprim domain |
| PF00749 | tRNA synthetases class I (E and Q) catalytic domain |
| PF09334 | tRNA synthetases class I (M) |
| PF00750 | tRNA synthetases class I (R) |
| PF01411 | tRNA synthetases class II (A) |
| PF03143 | Elongation factor Tu C-terminal domain |
| PF01026 | TatD related DNase |
| PF07973 | Threonyl and Alanyl tRNA synthetase second additional domain |
| PF03484 | tRNA synthetase B5 domain |
| PF00006 | ATP synthase alpha/beta family nucleotide-binding domain |
| PF00398 | Ribosomal RNA adenine dimethylase |
| PF07650 | KH domain |
| PF00571 | CBS domain |
| PF01300 | Telomere recombination |
| PF00575 | S1 RNA binding domain |
| PF02874 | ATP synthase alpha/beta family beta-barrel domain |
| PF00137 | ATP synthase subunit C |
| PF00448 | SRP54-type protein GTPase domain |
| PF01171 | PP-loop family |
| PF00306 | ATP synthase alpha/beta chain C terminal domain |
| PF13662 | Toprim domain |
| PF02272 | DHHA1 domain |
| PF01131 | DNA topoisomerase |
| PF03946 | Ribosomal protein L11 N-terminal domain |
| PF00117 | Glutamine amidotransferase class-I |
| PF00253 | Ribosomal protein S14p/S29e |
| PF02518 | Histidine kinase- DNA gyrase B- and HSP90-like ATPase |
| PF01588 | Putative tRNA binding domain |
| PF02978 | Signal peptide binding domain |
| PF01406 | tRNA synthetases class I (C) catalytic domain |
| PF02811 | PHP domain |
| PF02881 | SRP54-type protein helical bundle domain |

Table S2. Sequence Quality Scores for Acquired Genomes. For Each Data Source, Percent of Genomes Within Each Range of Genome Sequence Scores. Raw data and Statistics.

| **Sequence**  **Score** | **Complete** | **Draft** | **KBase** | **Patric** | **Broad** | **SRA** |
| --- | --- | --- | --- | --- | --- | --- |
| 0.1 | 0 | 0.4 | 0.2 | 0.4 | 0 | 12 |
| 0.2 | 0 | 1 | 0.6 | 0.9 | 0 | 12 |
| 0.3 | 0.04 | 2 | 1 | 1 | 0 | 12 |
| 0.4 | 0.1 | 3 | 2 | 2 | 0.08 | 15 |
| 0.5 | 0.07 | 5 | 3 | 4 | 0.5 | 15 |
| 0.6 | 0.1 | 9 | 7 | 7 | 1 | 13 |
| 0.7 | 0.3 | 11 | 9 | 9 | 2 | 13 |
| 0.8 | 0.4 | 18 | 16 | 16 | 8 | 7.2 |
| 0.9 | 0.4 | 27 | 22 | 22 | 38 | 0.54 |
| 1 | 99 | 23 | 39 | 37 | 51 | 0 |
| **Statistics** | | | | | | |
| Min | 0.2 | 0.03 | 0.04 | 0.03 | 0.4 | 0.01 |
| Max | 1.00 | 1.00 | 1.00 | 1.00 | 1.00 | 0.9 |
| Mean | 0.99 | 0.8 | 0.8 | 0.8 | 0.9 | 0.4 |
| N | 2672 | 12530 | 11923 | 11824 | 2381 | 11026 |
| SD | 0.042 | 0.2 | 0.18 | 0.19 | 0.08 | 0.22 |

Table S1. Number of Contigs per Genome. For Each Data Source, Percent of Genomes Within Each Range of Number of Contigs per Genome. Raw data and Statistics.

| **Count of Contigs** | **Complete** | **Draft** | **KBase** | **Patric** | **Broad** | **SRA** |
| --- | --- | --- | --- | --- | --- | --- |
| 1 | 60 | 0.6 | 13 | 12 | 0.2 | 0 |
| 2-5 | 33 | 2 | 8 | 8 | 5 | 0 |
| 6-10 | 4 | 4 | 4 | 3.9 | 6 | 0 |
| 11-50 | 1 | 29 | 24 | 24 | 60 | 0.6 |
| 51-100 | 0.4 | 22 | 17 | 17 | 21 | 3 |
| 101-500 | 0.7 | 34 | 29 | 30 | 7 | 50 |
| >500 | 0.07 | 8 | 5 | 6 | 0.5 | 46 |
| **Statistics** | | | | | | |
| Min | 1 | 1 | 1 | 1 | 1 | 25 |
| Max | 913 | 13915 | 13915 | 13915 | 1036 | 40069 |
| Mean | 5 | 190 | 130 | 151 | 48 | 1257 |
| N | 2672 | 12530 | 11923 | 11824 | 2381 | 11026 |
| SD | 30 | 419 | 327 | 408 | 66 | 2434 |

Table S2. Scores Based on Presence of 20 Standard tRNAs. For Each Data Source, Percent of Genomes Within Each Range of tRNA Scores. Raw data and Statistics.

| **tRNA Score** | **Complete** | **Draft** | **KBase** | **Patric** | **Broad** |
| --- | --- | --- | --- | --- | --- |
| 0.1 | 0.2 | 4 | 1 | 1.5 | 0.04 |
| 0.2 | 0.07 | 0.9 | 0.4 | 0.4 | 0 |
| 0.3 | 0.07 | 2 | 0.9 | 0.9 | 0 |
| 0.4 | 0.2 | 1 | 0.7 | 0.7 | 0.04 |
| 0.5 | 0.2 | 1 | 0.9 | 0.9 | 0.04 |
| 0.6 | 0.7 | 2 | 1.7 | 2 | 0 |
| 0.7 | 2 | 4 | 3 | 3 | 0.5 |
| 0.8 | 2 | 9 | 7 | 7 | 3 |
| 0.9 | 6 | 17 | 15 | 14 | 13 |
| 1 | 89 | 58 | 69 | 70 | 83 |
| **Statistics** | | | | | |
| Min | 0.1 | 0.1 | 0.1 | 0.1 | 0.1 |
| Max | 1 | 1 | 1 | 1 | 1 |
| Mean | 0.98 | 0.87 | 0.93 | 0.93 | 0.98 |
| N | 2672 | 12530 | 11753 | 11824 | 2381 |
| SD | 0.08 | 0.2 | 0.2 | 0.2 | 0.06 |

Table S3. Number of tRNA Predictions per Genome. For Each Data Source, Percent of Genomes Within Each Range of tRNA Count. Raw data and Statistics.

| **Count of tRNAs per Genome** | **Complete** | **Draft** | **KBase** | **Patric** | **Broad** |
| --- | --- | --- | --- | --- | --- |
| 0-40 | 23 | 27 | 21 | 21 | 2 |
| 41-50 | 27 | 23 | 25 | 25 | 17 |
| 51-60 | 21 | 19 | 22 | 22 | 31 |
| 61-70 | 11 | 13 | 14 | 14 | 24 |
| 71-100 | 15 | 16 | 17 | 16 | 26 |
| >100 | 3 | 1 | 2 | 2 | 0.5 |
| **Statistics** | | | | | |
| Min | 7 | 0 | 0 | 0 | 10 |
| Max | 173 | 284 | 185 | 236 | 284 |
| Mean | 55 | 53 | 55 | 55 | 62 |
| N | 2672 | 12530 | 11753 | 11824 | 2382 |
| SD | 19 | 20 | 19 | 19 | 14 |

Table S4. Number of Unique tRNA Anticodon Predictions per Genome. For Each Data Source, Percent of Genomes Within Each Range of Number of Unique tRNA Anticodons. Raw data and Statistics.

| **Count of**  **Unique**  **Anticodons** | **Complete** | **Draft** | **KBase** | **Patric** | **Broad** |
| --- | --- | --- | --- | --- | --- |
| 25 | 1 | 12 | 6 | 6 | 0.4 |
| 30 | 10 | 15 | 16 | 15 | 18 |
| 35 | 31 | 25 | 27 | 27 | 22 |
| 40 | 27 | 27 | 26 | 26 | 36 |
| 45 | 31 | 20 | 25 | 26 | 24 |
| >45 | 0.07 | 0.06 | 0.08 | 0.08 | 0 |
| **Statistics** | | | | | |
| Min | 0 | 0 | 0 | 0 | 0 |
| Max | 46 | 47 | 47 | 47 | 45 |
| Mean | 37 | 34 | 35 | 35 | 36 |
| N | 2674 | 12530 | 11753 | 11871 | 2383 |
| SD | 5 | 8 | 6 | 7 | 5 |

## tRNA Predictions by Anticodon

Of the 16 possible anticodons that start with 'A', 15 are relatively rare compared to other anticodons coding for the same amino acid. The exception is ACG, which codes for Arginine. Rows marked in YELLOW are anticodons that start with 'A'.

Table S5. tRNA Predictions by Anticodon. Count of the number of times tRNA anticodons were predicted in the genomes along with corresponding codon and amino acid.

| **#** | **Codon** | **Anticodon** | **Name** | **Count** |
| --- | --- | --- | --- | --- |
| 1 | GCU | AGC | Alanine | 44 |
| 2 | GCG | CGC | Alanine | 5041 |
| 3 | GCC | GGC | Alanine | 13554 |
| 4 | GCA | UGC | Alanine | 16568 |
| 5 | CGU | ACG | Arginine | 17108 |
| 6 | CGG | CCG | Arginine | 17086 |
| 7 | AGG | CCU | Arginine | 16479 |
| 8 | CGC | GCG | Arginine | 1143 |
| 9 | CGA | UCG | Arginine | 3574 |
| 10 | AGA | UCU | Arginine | 19257 |
| 11 | AAU | AUU | Asparagine | 30 |
| 12 | AAC | GUU | Asparagine | 18604 |
| 13 | GAU | AUC | Aspartate | 14 |
| 14 | GAC | GUC | Aspartate | 18352 |
| 15 | UGU | ACA | Cysteine | 8 |
| 16 | UGC | GCA | Cysteine | 18795 |
| 17 | GAG | CUC | Glutamate | 5411 |
| 18 | GAA | UUC | Glutamate | 17535 |
| 19 | CAG | CUG | Glutamine | 9458 |
| 20 | CAA | UUG | Glutamine | 18438 |
| 21 | GGU | ACC | Glycine | 13 |
| 22 | GGG | CCC | Glycine | 10770 |
| 23 | GGC | GCC | Glycine | 18057 |
| 24 | GGA | UCC | Glycine | 18367 |
| 25 | CAU | AUG | Histidine | 20 |
| 26 | CAC | GUG | Histidine | 18831 |
| 27 | AUU | AAU | Isoleucine | 35 |
| 28 | AUC | GAU | Isoleucine | 16772 |
| 29 | AUA | UAU | Isoleucine | 250 |
| 30 | CUU | AAG | Leucine | 2064 |
| 31 | UUG | CAA | Leucine | 18865 |
| 32 | CUG | CAG | Leucine | 13135 |
| 33 | CUC | GAG | Leucine | 16522 |
| 34 | UUA | UAA | Leucine | 17991 |
| 35 | CUA | UAG | Leucine | 18268 |
| 36 | AAG | CUU | Lysine | 9718 |
| 37 | AAA | UUU | Lysine | 18195 |
| 38 | AUG | CAU | Methionine | 19412 |
| 39 | UUU | AAA | Phenylalanine | 38 |
| 40 | UUC | GAA | Phenylalanine | 18603 |
| 41 | CCU | AGG | Proline | 111 |
| 42 | CCG | CGG | Proline | 9888 |
| 43 | CCC | GGG | Proline | 12184 |
| 44 | CCA | UGG | Proline | 18377 |
| 45 | UGA | UCA | SelCys | 5085 |
| 46 | AGU | ACU | Serine | 13 |
| 47 | UCU | AGA | Serine | 51 |
| 48 | UCG | CGA | Serine | 11909 |
| 49 | AGC | GCU | Serine | 18613 |
| 50 | UCC | GGA | Serine | 18464 |
| 51 | UCA | UGA | Serine | 18449 |
| 52 | UAG | CUA | Supres | 22 |
| 53 | UAA | UUA | Supres | 38 |
| 54 | ACU | AGU | Threonine | 312 |
| 55 | ACG | CGU | Threonine | 13052 |
| 56 | ACC | GGU | Threonine | 17543 |
| 57 | ACA | UGU | Threonine | 18436 |
| 58 | UGG | CCA | Tryptophan | 18522 |
| 59 | UAU | AUA | Tyrosine | 81 |
| 60 | UAC | GUA | Tyrosine | 18410 |
| 61 | GUU | AAC | Valine | 38 |
| 62 | GUG | CAC | Valine | 4928 |
| 63 | GUC | GAC | Valine | 13902 |
| 64 | GUA | UAC | Valine | 18210 |

Table S6. Genera with more than one genome with a rare tRNA. Count of Genomes in Genera with relatively rare tRNA Predictions.

| **Genus** | **Count** |
| --- | --- |
| *Bacillus* | 10 |
| *Bradyrhizobium* | 4 |
| *Butyrivibrio* | 28 |
| *candidate Division* | 4 |
| *Clostridiales* | 2 |
| *Clostridium* | 7 |
| *Coprococcus* | 2 |
| *Escherichia* | 3 |
| *Eubacterium* | 2 |
| *Fischerella* | 4 |
| *Flavobacterium* | 2 |
| *Fusobacterium* | 4 |
| *Gemmata* | 2 |
| *Klebsiella* | 4 |
| *Lachnospiraceae* | 9 |
| *Lactobacillus* | 150 |
| *Leuconostoc* | 21 |
| *Marine* | 3 |
| *Mesorhizobium* | 4 |
| *Mycobacterium* | 2 |
| *Mycoplasma* | 15 |
| *Oenococcus* | 14 |
| *Paenibacillus* | 2 |
| *Pantoea* | 2 |
| *Parvarchaeum* | 2 |
| *Portiera* | 3 |
| *Rhizobium* | 2 |
| *Salinispora* | 5 |
| *Salmonella* | 3 |
| *Serratia* | 13 |
| *Sphingobacterium* | 2 |
| *Spiroplasma* | 3 |
| *Staphylococcus* | 3 |
| *Streptococcus* | 66 |
| *Streptomyces* | 19 |
| *Vibrio* | 4 |
| *Weissella* | 7 |

## rRNA Scores - Percent in each range

Table S7. Scores Based on the Presence of a 23S, a 16S, and a 5S rRNA. For Each Data Source, Percent of Genomes Within Each Range of rRNA Scores. Raw data and Statistics.

| **rRNA Score** | **Complete** | **Draft** | **KBase** | **Patric** | **Broad** |
| --- | --- | --- | --- | --- | --- |
| 0.3 | 0.04 | 1 | 0.8 | 0.8 | 0.1 |
| 0.4 | 0.1 | 10 | 6 | 6 | 0.7 |
| 0.5 | 0.4 | 1 | 0.6 | 0.6 | 0.1 |
| 0.6 | 0.9 | 7 | 5 | 5 | 1 |
| 0.7 | 4 | 5 | 3 | 3 | 1 |
| 0.8 | 1 | 8 | 6 | 6 | 2 |
| 0.9 | 41 | 34 | 36 | 36 | 25 |
| 1 | 52 | 35 | 42 | 43 | 70 |
| **Statistics** | | | | | |
| Min | 0.3 | 0.3 | 0.3 | 0.3 | 0.3 |
| Max | 1 | 1 | 1 | 1 | 1 |
| Mean | 0.94 | 0.83 | 0.88 | 0.88 | 0.96 |
| N | 2667 | 12103 | 11492 | 11570 | 2382 |
| SD | 0.087 | 0.2 | 0.17 | 0.17 | 0.088 |

Table S8. Length of 23S rRNA Gene Predictions. For Each Data Source, Percent of Genomes Within Each Range of Length of 23S rRNAs. Raw data and Statistics.

| **Length**  **23S rRNA** | **Complete** | **Draft** | **KBase** | **Patric** | **Broad** |
| --- | --- | --- | --- | --- | --- |
| <=2800 | 3 | 18 | 12 | 12 | 13 |
| 2801-2850 | 3 | 3 | 3 | 3 | 3 |
| 2851-2900 | 34 | 32 | 33 | 33 | 17 |
| 2901-3000 | 46 | 41 | 43 | 43 | 61 |
| >3000 | 13 | 6 | 9 | 9 | 6 |
| **Statistics** | | | | | |
| Min | 1600 | 1400 | 1400 | 1400 | 1400 |
| Max | 9000 | 5700 | 9000 | 9000 | 5500 |
| Mean | 2900 | 2800 | 2900 | 2900 | 2900 |
| N | 10438 | 24187 | 28638 | 28627 | 9246 |
| SD | 200 | 310 | 280 | 280 | 400 |

Table S9. Length of 16S rRNA Gene Predictions. For Each Data Source, Percent of Genomes Within Each Range of Length of 16S rRNAs. Raw data and Statistics.

| **Length 16S rRNA** | **Complete** | **Draft** | **KBase** | **Patric** | **Broad** |
| --- | --- | --- | --- | --- | --- |
| <=1300 | 0.06 | 11 | 7 | 7 | 6 |
| 1301-1400 | 0.1 | 3 | 1 | 2 | 4 |
| 1401-1450 | 0.3 | 2 | 1 | 1 | 2 |
| 1451-1500 | 16 | 14 | 14 | 14 | 10 |
| 1501-1550 | 78 | 68 | 72 | 73 | 76 |
| >1550 | 6 | 2 | 3 | 3 | 2 |
| **Statistics** | | | | | |
| Min | 1100 | 740 | 740 | 740 | 760 |
| Max | 3500 | 3300 | 4300 | 4300 | 3300 |
| Mean | 1500 | 1500 | 1500 | 1500 | 1500 |
| N | 10456 | 25064 | 28994 | 29010 | 9039 |
| SD | 110 | 180 | 160 | 160 | 190 |

Table S10. Length of 5S rRNA Gene Predictions. For Each Data Source, Percent of Genomes Within Each Range of Length of 5S rRNAs. Raw data and Statistics.

| **Length 5S rRNA** | **Complete** | **Draft** | **KBase** | **Patric** | **Broad** |
| --- | --- | --- | --- | --- | --- |
| <=100 | 0.03 | 5 | 4 | 4 | 4 |
| 101-110 | 5 | 6 | 6 | 5 | 5 |
| 111-115 | 87 | 86 | 86 | 86 | 88 |
| 116-120 | 6 | 3 | 3 | 3 | 2 |
| 121-130 | 0.1 | 0.05 | 0.05 | 0.03 | 0.008 |
| >130 | 0.02 | 0.01 | 0.007 | 0.01 | 0.008 |
| **Statistics** | | | | | |
| Min | 97 | 74 | 74 | 74 | 76 |
| Max | 200 | 220 | 220 | 220 | 180 |
| Mean | 110 | 110 | 110 | 110 | 110 |
| N | 10796 | 39522 | 40855 | 40649 | 12347 |
| SD | 4.7 | 5.9 | 5.8 | 5.8 | 4.5 |

Table S11. Number of 23S Predictions per Genome. For Each Data Source, Percent of Genomes Within Each Range of Number of 23S rRNAs. Raw data and Statistics.

| **Count 23S rRNA** | **Complete** | **Draft** | **KBase** | **Patric** | **Broad** |
| --- | --- | --- | --- | --- | --- |
| 0-1 | 20 | 70 | 58 | 58 | 23 |
| 2-3 | 33 | 10 | 16 | 16 | 22 |
| 4-6 | 30 | 13 | 17 | 17 | 42 |
| 7-9 | 13 | 5 | 7 | 6 | 13 |
| 10-12 | 4 | 1 | 2 | 2 | 0.5 |
| >12 | 1 | 0.07 | 0.9 | 0.9 | 0 |
| **Statistics** | | | | | |
| Min | 0 | 0 | 0 | 0 | 0 |
| Max | 15 | 19 | 19 | 19 | 11 |
| Mean | 3.9 | 2 | 2.5 | 2.5 | 3.9 |
| N | 2667 | 12103 | 11492 | 11570 | 2382 |
| SD | 2.7 | 2.4 | 2.6 | 2.6 | 2.3 |

Table S12. Average Predicted Gene Length per Genome. For Each Data Source, Percent of Genomes Within Each Range of Average Gene Length in each Genome. Raw data and Statistics.

| **Average Gene Length** | **Complete** | **Draft** | **KBase** | **Patric** | **Broad** |
| --- | --- | --- | --- | --- | --- |
| <=600 | 0.2 | 0.7 | 0.4 | 0.7 | 0 |
| 601-700 | 0.3 | 1 | 0.8 | 0.9 | 0.04 |
| 701-800 | 3 | 4 | 3 | 3 | 0.04 |
| 801-900 | 23 | 33 | 32 | 31 | 17 |
| 901-1000 | 54 | 54 | 54 | 54 | 77 |
| >1000 | 19 | 7 | 9 | 9 | 6 |
| **Statistics** | | | | | |
| Min | 560 | 230 | 260 | 230 | 630 |
| Max | 1200 | 1300 | 1200 | 1300 | 1200 |
| Mean | 940 | 910 | 920 | 920 | 940 |
| N | 2671 | 12530 | 11340 | 11821 | 2381 |
| SD | 79 | 78 | 76 | 80 | 51 |

Table S13. Average Predicted Gene Density per Genome. For Each Data Source, Percent of Genomes Within Each Range of Average Gene Density (genes per kilobase) in each Genome. Raw data and Statistics.

| **Average Density** | **Complete** | **Draft** | **KBase** | **Patric** | **Broad** |
| --- | --- | --- | --- | --- | --- |
| 0-.8 | 3 | 0.8 | 2 | 2 | 1 |
| 0.81-.9 | 26 | 14 | 17 | 16 | 12 |
| 0.91-1.0 | 55 | 64 | 64 | 65 | 85 |
| 1.01-1.1 | 11 | 16 | 14 | 14 | 2 |
| 1.11-1.2 | 4 | 3 | 2 | 2 | 0 |
| >1.2 | 0.5 | 2 | 1.0 | 1 | 0.04 |
| **Statistics** | | | | | |
| Min | 0.42 | 0.69 | 0.42 | 0.42 | 0.75 |
| Max | 1 | 3 | 3 | 3 | 1 |
| Mean | 0.94 | 0.97 | 0.95 | 0.96 | 0.92 |
| N | 2671 | 12530 | 11340 | 11821 | 2381 |
| SD | 0.08 | 0.11 | 0.08 | 0.1 | 0.04 |

Table S14. Scores Based on the Presence of 102 Essential Genes. For Each Data Source, Percent of Genomes Within Each Range of Essential Gene Score Assigned to Genomes. Raw data and Statistics.

| **Essential Gene Score** | **Complete** | **Draft** | **KBase** | **Patric** | **Broad** |
| --- | --- | --- | --- | --- | --- |
| 0.1 | 0.04 | 0.1 | 0.05 | 0.02 | 0.042 |
| 0.2 | 0 | 0.1 | 0.04 | 0.02 | 0 |
| 0.3 | 0 | 0.3 | 0.04 | 0.03 | 0 |
| 0.4 | 0 | 0.4 | 0.09 | 0.1 | 0 |
| 0.5 | 0.07 | 0.4 | 0.1 | 0.1 | 0 |
| 0.6 | 0.1 | 0.5 | 0.3 | 0.3 | 0 |
| 0.7 | 0.1 | 0.7 | 0.3 | 0.4 | 0 |
| 0.8 | 0.2 | 0.8 | 0.4 | 0.5 | 0 |
| 0.9 | 0.6 | 1 | 1 | 1 | 0.04 |
| 1 | 99 | 96 | 98 | 97 | 100 |
| **Statistics** | | | | | |
| Min | 0.1 | 0 | 0.03 | 0.05 | 0.9 |
| Max | 1 | 1 | 1 | 1 | 1 |
| Mean | 0.99 | 0.98 | 0.99 | 0.99 | 1 |
| N | 2667 | 12528 | 11742 | 11821 | 2382 |
| SD | 0.03 | 0.09 | 0.05 | 0.05 | 0.003 |

Table S15. Scores Based on the Combined Total Score for Each Genome. For Each Data Source, Percent of Genomes Within Each Range of Total Score Assigned to Genomes. Raw data and Statistics.

| **Total**  **Score** | **Complete** | **Draft** | **KBase** | **Patric** | **Broad** |
| --- | --- | --- | --- | --- | --- |
| 0.1 | 0 | 0 | 0.008 | 0.008 | 0 |
| 0.2 | 0 | 0.2 | 0.04 | 0.1 | 0.04 |
| 0.3 | 0.07 | 0.6 | 0.2 | 0.5 | 0 |
| 0.4 | 0 | 1 | 0.2 | 0.3 | 0 |
| 0.5 | 0 | 3 | 0.8 | 1 | 0 |
| 0.6 | 0.2 | 3 | 2 | 2 | 0 |
| 0.7 | 0.4 | 5 | 3 | 3 | 0.2 |
| 0.8 | 1 | 13 | 10 | 10 | 2 |
| 0.9 | 5 | 29 | 24 | 24 | 8 |
| 1 | 93 | 45 | 60 | 59 | 91 |
| **Statistics** | | | | | |
| Min | .3 | .1 | .1 | .1 | .1 |
| Max | 1 | 1 | 1 | 1 | 1 |
| Mean | 0.97 | 0.85 | 0.9 | 0.89 | 0.96 |
| N | 2674 | 12518 | 11898 | 11864 | 2383 |
| SD | 0.05 | 0.1 | 0.1 | 0.1 | 0.05 |

Table S18. Number of Genomes, Average Score, and Standard Deviation by Sequencing Technology

| **Technology** | **Number** | **Average Score** | **Standard Deviation** |
| --- | --- | --- | --- |
| Sanger | 148 | 0.86 | 0.13 |
| Illumina | 9466 | 0.86 | 0.15 |
| 454 | 3028 | 0.9 | 0.08 |
| Ion Torrent | 151 | 0.85 | 0.09 |
| PacBio | 50 | 0.98 | 0.02 |
| SOILD | 37 | 0.67 | 0.19 |
| Sanger; Illumina | 28 | 0.92 | 0.09 |
| Sanger; 454 | 247 | 0.91 | 0.11 |
| Sanger; 454; Illumina | 118 | 0.92 | 0.06 |
| 454; Illumina | 1350 | 0.93 | 0.09 |
| 454; Ion Torrent | 27 | 0.86 | 0.07 |
| 454; Illumina; PacBio | 12 | 0.96 | 0.05 |
| Illumina; PacBio | 62 | 0.97 | 0.04 |
| 454; PacBio | 6 | 0.97 | 0.02 |
| 454; SOILD | 11 | 0.94 | 0.07 |
| Other | 135 | 0.79 | 0.23 |

Table S19. Number of Genomes, Average Score, and Standard Deviation by Assembler Used

| **Assembler** | **Number** | **Average Score** | **Standard Deviation** |
| --- | --- | --- | --- |
| A5 | 73 | 0.84 | 0.07 |
| ABySS | 88 | 0.9 | 0.05 |
| Allpaths | 2015 | 0.94 | 0.1 |
| Allpaths; Velvet | 755 | 0.75 | 0.24 |
| Arachne | 13 | 0.79 | 0.12 |
| CLC | 48 | 0.83 | 0.10 |
| CLC Bio | 586 | 0.87 | 0.10 |
| CLC Bio; GS Assembler; GS DeNovo | 8 | 0.94 | 0.05 |
| CLC De Novo | 9 | 0.88 | 0.04 |
| Celera | 2057 | 0.86 | 0.12 |
| Consed; GS Assembler; GS DeNovo | 10 | 0.85 | 0.001 |
| Edena | 33 | 0.85 | 0.05 |
| GS Assembler | 24 | 0.81 | 0.11 |
| GS Assembler; GS DeNovo | 153 | 0.88 | 0.09 |
| Geneious | 8 | 0.87 | 0.11 |
| HGAP | 24 | 0.99 | 0.01 |
| MIRA | 136 | 0.90 | 0.07 |
| Newbler | 2546 | 0.88 | 0.11 |
| Newbler; CLC Bio | 40 | 0.86 | 0.067 |
| Newbler; MIRA | 7 | 0.94 | 0.04 |
| Newbler; Phrap | 23 | 0.98 | 0.02 |
| Newbler; SOAP | 12 | 0.89 | 0.08 |
| Newbler; Velvet | 109 | 0.89 | 0.14 |
| Other | 152 | 0.82 | 0.15 |
| Phrap | 30 | 0.93 | 0.12 |
| Phrap; MIRA | 6 | 0.95 | 0.01 |
| Phrap; SOAP | 7 | 0.83 | 0.19 |
| Ray | 16 | 0.85 | 0.08 |
| SMRT | 11 | 0.98 | 0.01 |
| SOAP | 652 | 0.67 | 0.20 |
| SPAdes | 16 | 0.91 | 0.04 |
| SeqMan | 27 | 0.92 | 0.07 |
| Unknown | 462 | 0.84 | 0.13 |
| Velvet | 1724 | 0.81 | 0.12 |
| Velvet; AMOScmp | 13 | 0.92 | 0.06 |
| Velvet; CLC Bio | 7 | 0.91 | 0.05 |
| Velvet; Geneious | 8 | 0.89 | 0.13 |
| Velvet; SOAP | 10 | 0.94 | 0.02 |
| in house | 65 | 0.94 | 0.02 |

Table S20. Comparison of the Percent of Genomes of Selected Genera Between the Entire Database and the Broad

| **Genus** | **Entire Database** | **Broad** |
| --- | --- | --- |
| *Escherichia* | 10.3 | 23.6 |
| *Enterococcus* | 5.2 | 19.7 |
| *Staphylococcus* | 4.6 | 9.4 |
| *Brucella* | 3.0 | 8.7 |
| *Acinetobacter* | 2.9 | 5.3 |
| *Mycobacterium* | 3.1 | 5.6 |
| *Bacillus* | 2.8 | 4.1 |
| *Pseudomonas* | 2.3 | 3.8 |
