# Supplementary material for: Quality scores for 32,000 genomes
Source: Stand Genomic Sci. 2014 Dec 8;9:20. doi: 10.1186/1944-3277-9-20 (PMC4334873; doi:10.1186/1944-3277-9-20)
Supplement: Additional file 2 — Additional figures mentioned in the text with bar charts used to visually support the analysis. [file 1944-3277-9-20-S2.doc]

**Additional file 2: Figures**


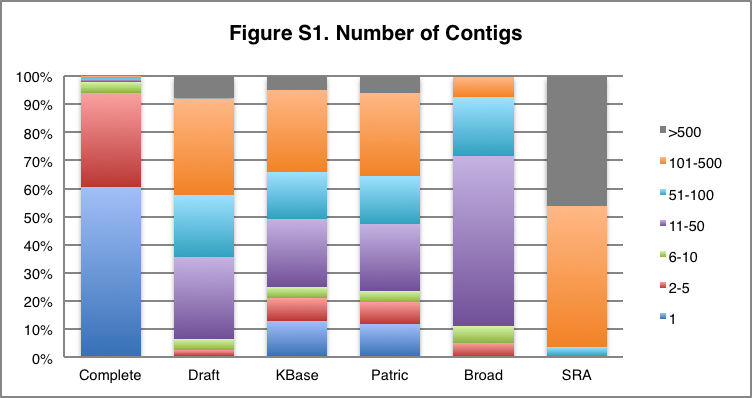


Figure S1. Number of Contigs Per Genome. For Each Data Source, Percent of Genomes Within Each Range of Number of Contigs per Genome.


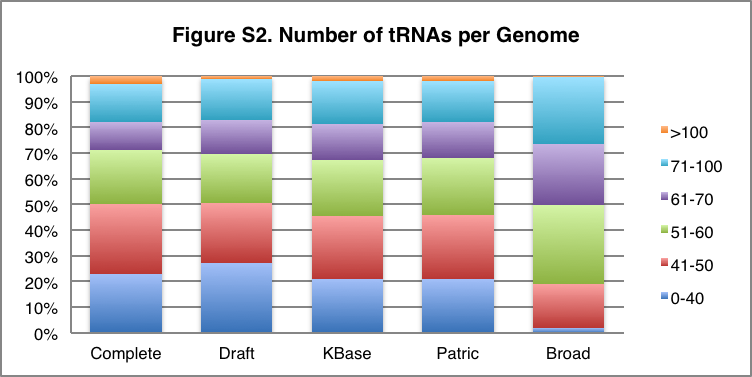


Figure S2. Number of tRNA Predictions per Genome. For Each Data Source, Percent of Genomes Within Each Range of tRNA Count.


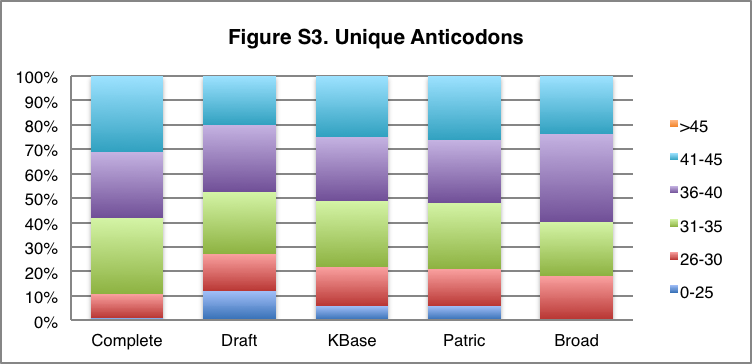


Figure S3. Number of Unique Anticodons per Genome. For Each Data Source, Percent of Genomes Within Each Range of Number of Unique tRNA Anticodons.


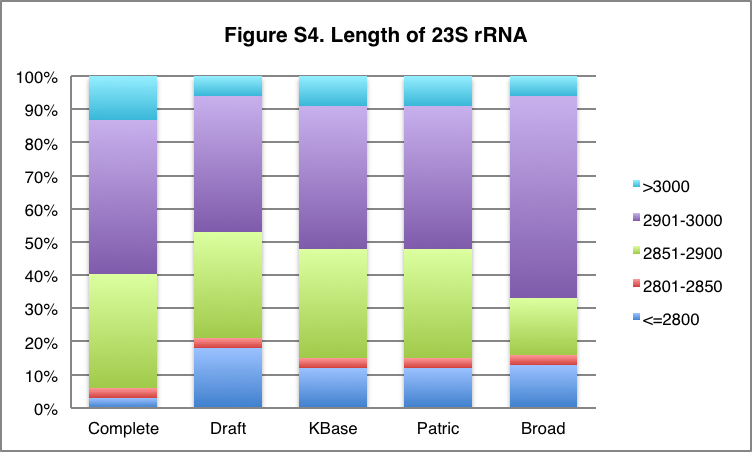


Figure S4. Length of 23S rRNA Predictions. For Each Data Source, Percent of Genomes Within Each Range of Length of 23S rRNAs.


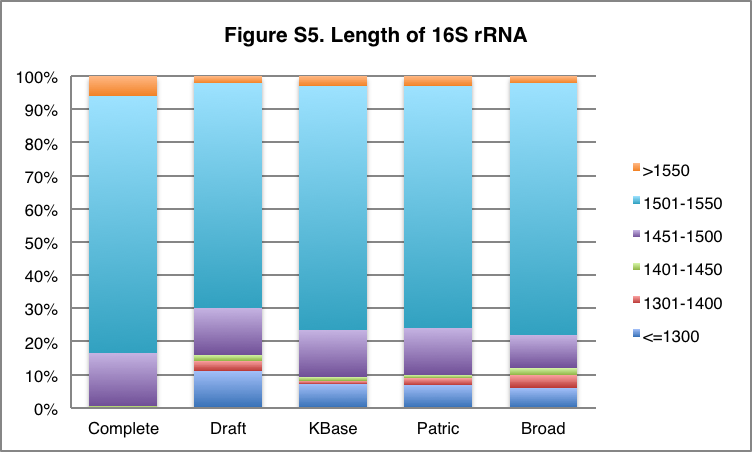


Figure S5. Length of 16S rRNA Predictions. For Each Data Source, Percent of Genomes Within Each Range of Length of 16S rRNAs.


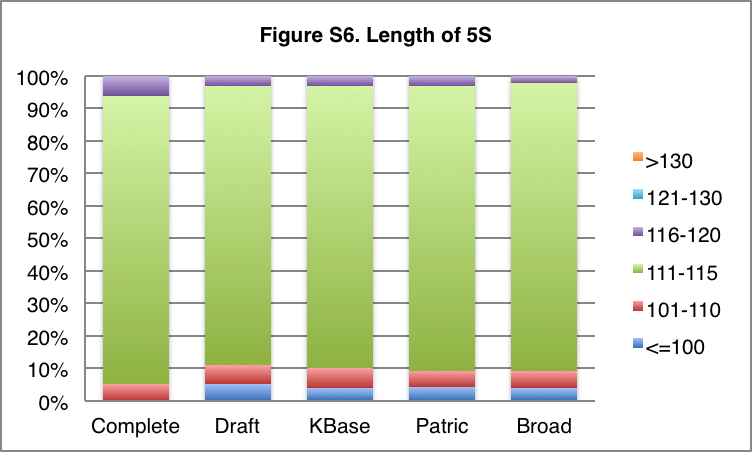


Figure S6. Length of 5S rRNA Predictions. For Each Data Source, Percent of Genomes Within Each Range of Length of 5S rRNAs.


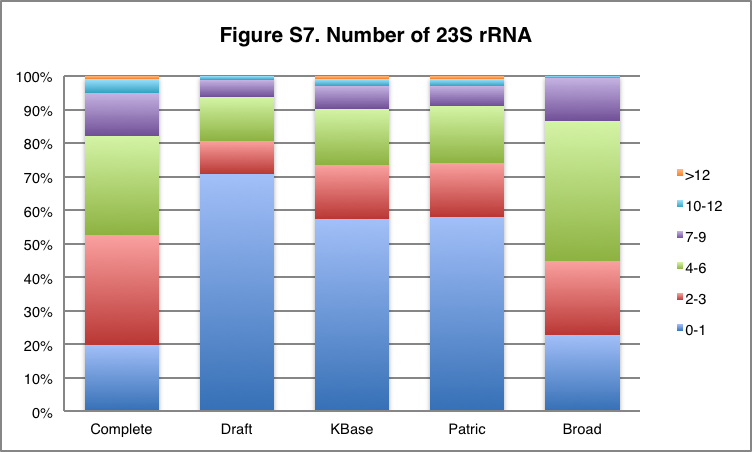


Figure S7. Number of 23S rRNA Predictions per Genome. For Each Data Source, Percent of Genomes Within Each Range of Number of 23S rRNAs.


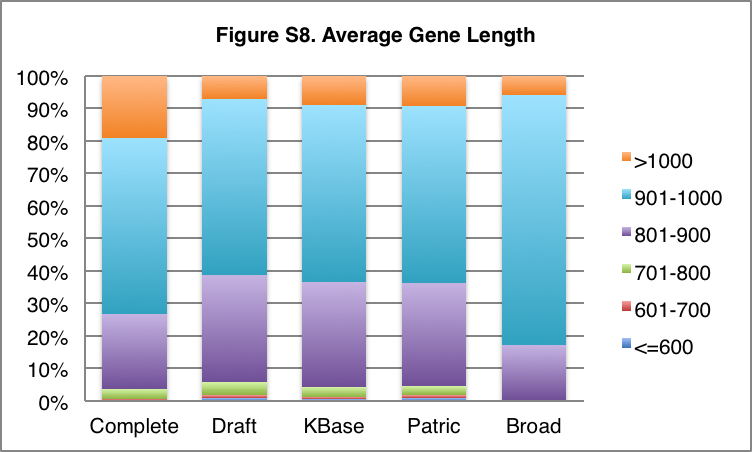


Figure S8. Average Predicted Gene Length by Genome. For Each Data Source, Percent of Genomes Within Each Range of Average Gene Length.


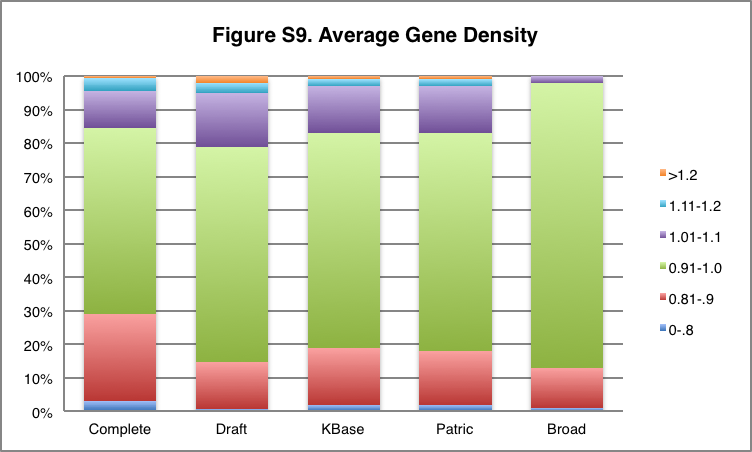


Figure S9. Average Predicted Gene Density for Genomes. For Each Data Source, Percent of Genomes Within Each Range of Average Gene Density (genes per kilobase).


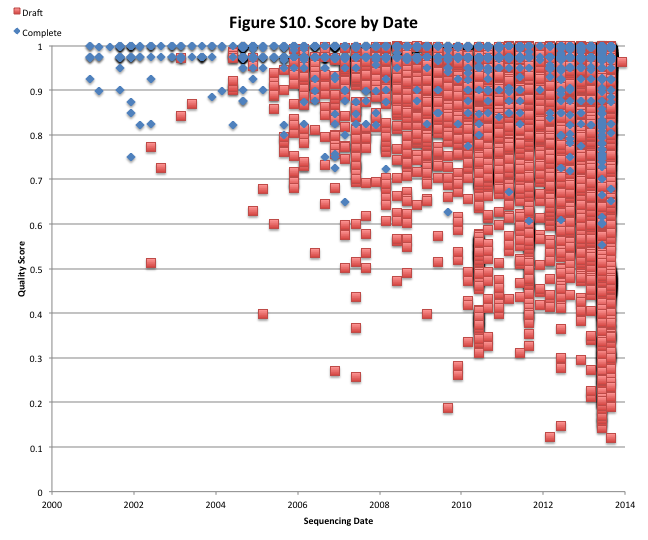


Figure S10. Quality Scores by Available Sequencing Date. The sequencing date was parsed from GenBank complete and draft files. All dates within a quarter were assigned to the centroid date of the quarter. Points are color coded by the ‘complete’ (blue) or ‘draft’ (red) designation with ‘complete’ plotted second and on top of ‘draft’ points.


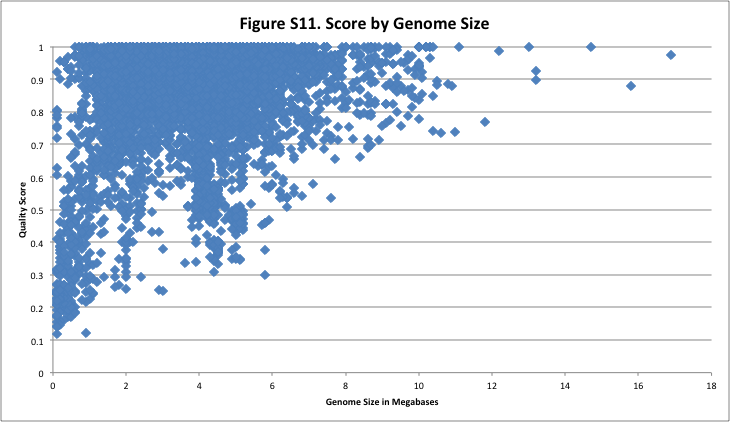
 Figure S11. Quality Scores by Genome Size. Genome size was rounded to megabases and plotted against the assigned quality score. Includes all data.


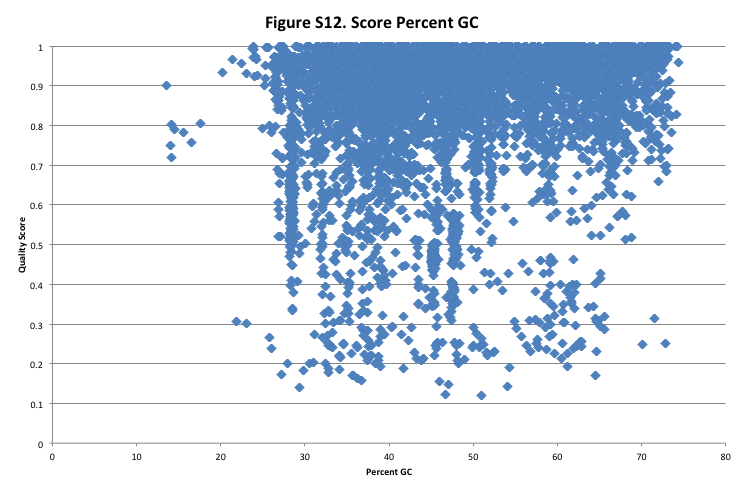
 Figure S12. Quality Scores by Percent GC Content. The percent GC of the genomes was plotted against the quality scores. Includes all data.
